# Dietary *Enterococcus faecalis* LAB31 Improves Growth Performance, Reduces Diarrhea, and Increases Fecal *Lactobacillus* Number of Weaned Piglets

**DOI:** 10.1371/journal.pone.0116635

**Published:** 2015-01-24

**Authors:** Yuanliang Hu, Yaohao Dun, Shenao Li, Dongxiao Zhang, Nan Peng, Shumiao Zhao, Yunxiang Liang

**Affiliations:** 1 State Key Laboratory of Agricultural Microbiology, College of Life Science and Technology, Huazhong Agricultural University, Wuhan 430070, P.R. China; 2 College of Life Sciences, Hubei Normal University, Huangshi, Hubei 435002, P.R. China; GI Lab, UNITED STATES

## Abstract

Lactic acid bacteria (LAB) have been shown to enhance performance of weaned piglets. However, few studies have reported the addition of LAB *Enterococcus faecalis* as alternatives to growth promoting antibiotics for weaned piglets. This study evaluated the effects of dietary *E. faecalis* LAB31 on the growth performance, diarrhea incidence, blood parameters, fecal bacterial and *Lactobacillus* communities in weaned piglets. A total of 360 piglets weaned at 26 ± 2 days of age were randomly allotted to 5 groups (20 pens, with 4 pens for each group) for a trial of 28 days: group N (negative control, without antibiotics or probiotics); group P (Neomycin sulfate, 100 mg/kg feed); groups L, M and H (supplemented with *E. faecalis* LAB31 0.5×10^9^, 1.0×10^9^, and 2.5×10^9^ CFU/kg feed, respectively). Average daily gain and feed conversion efficiency were found to be higher in group H than in group N, and showed significant differences between group H and group P (*P_0_* < 0.05). Furthermore, groups H and P had a lower diarrhea index than the other three groups (*P_0_* < 0.05). Denaturing gradient gel electrophoresis (DGGE) showed that the application of probiotics to the diet changed the bacterial community, with a higher bacterial diversity in group M than in the other four groups. Real-time PCR revealed that the relative number of *Lactobacillus* increased by addition of probiotics, and was higher in group H than in group N (*P_0_* < 0.05). However, group-specific PCR-DGGE showed no obvious difference among the five groups in *Lactobacillus* composition and diversity. Therefore, the dietary addition of *E. faecalis* LAB31 can improve growth performance, reduce diarrhea, and increase the relative number of *Lactobacillus* in feces of weaned piglets.

## Introduction

Nursery piglets often face post-weaning challenges such as diarrhea, low feed intake, and weight loss, which can cause serious damages to intestinal health and function [1]. Usually, growth promoting antibiotics (sub-therapeutic doses to animal feed) are used to reduce the weaning stress and improve growth performance of piglets in many countries. However, the application of antibiotics in animal husbandry increases the resistance of pathogenic bacteria to antibiotics. Moreover, antibiotic use in animals may contribute to the emergence of antibiotic resistance bacteria in humans, due to the antibiotic-resistant bacteria and genes can spread from animals to humans through food chains [2], [3]. Therefore, since 2006, European Union legislation has banned the use of antibiotics as growth promoters in animal feeds, and the banning of their use is under consideration in more countries due to the potential increase of antibiotic resistant bacteria [4].

Probiotics, defined as living microbes that confer a health effect on the host when consumed in adequate amounts, may serve as an alternative for antibiotics to promote the growth of weaned piglets [5]. The bacteria used as probiotics are mainly lactic acid bacteria (LAB) and *Bacillus*. LAB are a group of Gram-positive bacteria, which excrete lactic acid as a main product in sugar fermentation [6]. Some LAB including *Enterococcus faecium*, *Bifidobacterium bifidum* and several *Lactobacillus* species are the most common probiotics added to food or feed products [7], [8]. The most common species of *Enterococcus* spp. are *E. faecium* and *E. faecalis*, which can be isolated from a wide variety of habitats, such as the gastrointestinal tract, the oral cavity, and the vagina in animal as normal commensals [9], [10]. In pig production, *E. faecium* strains are frequently used and has recently been shown to decrease the incidence of diarrhea and the count of *E. coli*, and improve animal performance, and feed conversion [11], [12], [13]. Moreover, *E. faecalis* strains have potential benefits for host to initiate pro-inflammatory responses in the disease-susceptible [14], and have a high capacity to respond to certain microbes by secreting high levels of inflammatory cytokines [15]. The administrations of *E. faecalis* strains were observed to improve constipation [16] and villous atrophy [17].

Nowadays, it is increasingly realized that gastrointestinal health is influenced by the composition of the microbial community [18], [19]. The intestinal microbiota balance of pigs can be modulated by dietary intervention [12], [13]. Within this regard, enhancing the beneficial members of the gastrointestinal tract ecosystem, for instance *Lactobacillus*, by supplying specific dietary ingredients such as probiotics, has proven to be an appropriate option [20]. Furthermore, intestinal microbiota has been found to be an important factor related to energy metabolism [21], amino acid turnover [22], bacterial pathogen colonization and health of pigs [18].

However, most studies are focused on the *E. faecium* strains, and few reports are available about the application of the *E. faecalis* strains as an alternative to growth promoting antibiotics for piglets. In the present work, we investigated the effects of *E. faecalis* LAB31 on the growth performance, diarrhea incidence, blood parameters and fecal bacterial community of weaned piglets. Furthermore, the copy number and community of *Lactobacillus* in feces were analyzed by real-time PCR and denaturing gradient gel electrophoresis (DGGE), respectively.

## Materials and Methods

### Probiotics

The *E. faecalis* LAB31 (CGMCC No. 4848) was isolated from fresh swine feces, and certified as a feed additive by the Ministry of Agriculture of China. The commercial product (the viable count of 2.5×10^9^ CFU/g), which was cultured by solid state fermentation and made into dry powder, was donated by Gold-Tide Biotechnology CO., LTD (Beijing, China).

### Animal and Experimental Design

At weaning, a total of 360 Duroc × Landrace × Yorkshire piglets, with an age of 26 ± 2 d and a similar weight from 80 litters were selected for a trial of 28 days. All procedures and the use of animals in this experiment were carried out in accordance with the guidelines issued by Huazhong Agricultural University Animal Care and Use Committee, and Huazhong Agricultural University Animal Care and Use Committee specifically approved this study.

All piglets were divided into 20 pens with 18 piglets in each pen (male: female, 1:1; body weight of 7.66 ± 0.54 kg). According to a randomized block design, the piglets were assigned into 5 groups: Negative control (group N, without antibiotics or probiotics); positive control (group P, neomycin sulfate, 100 mg/kg feed); and experimental groups (L, M, and H with *E. faecalis* LAB31 0.5×10^9^, 1.0×10^9^ and 2.5×10^9^ CFU/kg feed, respectively), and each group had 4 replicates. The trial was divided into 2 phases: phase I (d 1 to d 14 post weaning) and phase II (d 15 to d 28). Throughout the experiment, the piglets were housed in a temperature-controlled nursery room (25 ± 2°C), and feed and water were available ad libitum. The diets (S1 Table) were formulated according to NRC recommendations [23]. All piglets were vaccinated against pseudorabies, foot-and-mouth disease, circovirus, blue ear disease, asthma, and hog cholera.

### Measurements and Sampling

Body weight and feed consumption were measured at 7:00 a.m. on day 1, 15, and 29 to determine average daily gain (ADG), average daily feed intake (ADFI) and ratio of ADG to ADFI (G/F). The fecal score was recorded twice a day by using a four-grade system (0 to firm, dry, normal consistency of feces formed; 1 to pasty; 2 to thick and fluid; and 3 to watery) [24]. Diarrhea was defined as the daily sum score of two equal to or greater than 2. The incidence of diarrhea (%) was calculated by the total number of diarrheal piglets over a period divided by the number of piglets and days in that period multiplied by 100. On day 27, the fasting blood samples (5 ml) were collected from 5 piglets in each group via jugular vein into EDTA-K_2_ disodium vacuum tubes (Jiangxi Hongda Group, Jinxian, China). The leukocyte, erythrocyte, platelet count, hemoglobin concentration, mean corpuscular hemoglobin concentration (MCHC), and lymphocyte (%) in whole blood were measured the same day using an automated hematology analyzer CEll-DYN 3700 (Abbott, Chicago, USA). On day 28, fecal samples (5 piglets per group, at least 1 piglet per pen) were collected by rectal massage and then stored at −80°C before use.

### DNA Extraction and PCR-DGGE

The DNA from the fecal samples was extracted using the QIAamp DNA Stool Mini Kit (Qiagen, Duesseldorf, Germany) according to the manufacturer’s instructions, using 95°C for the initial lysis step [25]. For elution, the matrix-DNA complex was resuspended in 100 µl elution buffer, and stored at-20°C before use. DNA concentration and quantity were tested on a Nanodrop ND-100 spectrophotometer (Thermo, Wilmington, USA).

The primers 968F-GC and 1401R (Table 1; Invitrogen, Shanghai, China) targeted to the V6~V8 regions of 16S rDNA were used for total bacteria PCR. Amplification was carried out using a Bio-Rad T100 Thermal Cycler (Bio-Rad, Hercules, USA). The 50 µl reaction mixture consisted of 5 µl of 10 × PCR buffer (Takara, Dalian, China), 2 µl of dNTPs (2.5 mM of each, Takara), 1 µl of each primer (10 µM), 2.5 U of Taq polymerase (Takara) and 50 ng template DNA. The amplification program was 1 cycle for 7 min at 94°C, 35 cycles of (94°C for 30 s, 56°C for 20 s, and 68°C for 30 s) and a final elongation of 7 min at 68°C. The PCR products from each treatment (5 piglets) were mixed together as one product. Before running on DGGE gels, the products were cleaned using a PCR Purification Kit (Omega, Norcross, USA).

**Table 1:** Primers used for DGGE and qPCR.

**Target group**	**Prime Sequence (5′→3′)**	**Amplicon size (bp)**	**Annealing temp (°C)**	**Reference**
Total bacteria DGGE	AACGCGAAGAACCTTAC (968F-GC*)	435	56	[28]
	CGGTGTGTACAAGACCC (1401R)			
*Lactobacillus* DGGE	AGCAGTAGGGAATCTTCCA (Lac1)	380	61	[29]
	ATTYCACCGCTACACATG (Lac2-GC*)			
Total bacteria	CGGYCCAGACTCCTACGGG	200	60	[30]
	TTACCGCGGCTGCTGGCAC			
*Lactobacillus*	CGATGAGTGCTAGGTGTTGGA	186	60	[31]
	CAAGATGTCAAGACCTGGTAAG			

*GC clamp (5′-CGCCCGGGGCGCGCCCCGGGCGGCCCGGGGGCACCGGGGG-3′)

Gels containing 8% (w/v) polyacrylamide (acrylamide/bisacrylamide, 37.5/1) were formed using a Model 475 Gradient Delivery System (Bio-Rad) in 0.5 × Tris-acetate-EDTA (TAE) buffer (pH 8.0) with a 42% to 58% urea-formamide denaturing gradient (100% corresponding to 7M urea and 40% deionized formamide). The gels formed were allowed to polymerize for 4 h before use. DGGE was performed using the DCode Universal Mutation Detection System (Bio-Rad) by electrophoresing 10 µl of PCR product (32 ng/µl, plus 10 µl loading buffer) at 10 V for 10 min and subsequently at 85 V for 16 h in 0.5 × TAE buffer at 60℃. The DNA bands in gels were visualized by silver staining [26].

The PCR-DGGE for *Lactobacillus* was performed as described above with the following modifications. The primers were replaced by Lac1 and Lac2-GC (Table 1; 10 µM; Invitrogen). The PCR program was 1 cycle at 94°C for 7 min; 35 cycles of 94°C for 30 s, 61°C for 1 min, and 68°C for 1 min; and a final extension at 68°C for 7 min. The denaturant gradient range of gel was from 41% to 60% with 10 µl of PCR product (24 ng/µl) electrophoresed at 85 V for 16 h.

### DGGE Bands Sequencing and Analyses

Quantity One (version 4.6.2; Discovery Series, Bio-Rad) was used for band analysis. The number of bands detected in a DGGE lane was used as a measure of the number of species present (species richness, S). Sample indexes were calculated based on gauss trace quality, which was determined using background subtraction as an estimate of the relative population size of each species. Shannon index (H′), Evenness (J), and Simpson index (1/D) [27] were calculated using the software BIO-DAP (Fundy National Park, Canada).

In order to identify the species represented by DGGE analysis, DNA from bands was sequenced. Bands representing the range of migration patterns on the gels were selected and bands that migrated to the same position in different lanes were checked for similarity. To diffuse DNA from the gels into the water before sequencing, DGGE bands were excised and stored in 50 µl of water at 4°C for 16~24 h. Subsequently, 5 µl of DNA solution was used to re-amplify the DNA using the same reaction mixture and condition as previously described except that the primer without the GC clamp was used. PCR products were cleaned using a PCR Purification Kit (Omega) and then sequenced (Invitrogen, Shanghai, China). For identification of the closest relatives, newly determined sequences were compared with those available in the V6–V8 regions of the 16S rDNA sequences of the GenBank DNA database (www.ncbi.nih.gov). The identities of the relatives were determined on the basis of the highest score.

### Quantitative Real-time PCR Analysis of Fecal Total Bacteria and *Lactobacillus* spp.

The quantitative real-time PCR (qPCR) was performed to quantify the total bacteria and *Lactobacillus* spp. counts by using previously described primers and annealing temperatures (Table 1). The amplification was performed using an iTaq Universal SYBR Green Supermix (Bio-Rad) in an Applied Biosystems ViiA 7 Real-Time PCR System (ABI) as follows: 1 cycle at 95°C for 10 min, followed by 40 cycles (95°C for 30 s, 50 to 60°C for 20 s and 72°C for 30 s), and the data were collected at the extension step (72°C for 30 s). Standard curves were generated using serial dilutions of purified genomic DNA obtained by standard PCR using the corresponding primers. Melting curves were checked after amplification to ensure the accuracy of the amplification results. The results were expressed in log_10_ gene copy number/g feces (wet weight), whereas the values for the *Lactobacillus* spp. were expressed in relative numbers versus total bacteria.

### Statistical Analyses

Data of growth performance and diarrhea ratio were analyzed according to a randomized complete block design using the GLM procedure of SAS (version 8.0; SAS Inst. Inc., Cary, NC), with pen as the experimental unit. The parameters of hematological traits and bacterial copy numbers were analyzed based on variances using the same SAS software, with piglet as the experimental unit (5 piglets). The copy numbers of total bacteria were transformed (log_10_) before statistical analysis, and the percent of *Lactobacillus* was calculated before transformation to logarithmic form. Statistical differences among treatments were separated by Duncan’s multiple range tests and considered significant at *P* values < 0.05. The linear and quadratic effect of 4 different doses of probiotics (groups N, L, M, and H) were analysed by SAS software, and considered significantly correlation at *P* values < 0.05.

## Results

### Growth Performance and Diarrhea Incidence

Throughout the experiment, only 1 piglet in group L suddenly died with suspected edema disease. During phase I, a significant improvement in ADG and G/F was observed in the 4 groups (L, M, H and P) supplemented with probiotics or antibiotics as compared to group N (*P_0_* < 0.05), but with no significant differences among the 4 groups (Table 2). During phase II, a higher ADG and G/F was found in groups P and H than in group N (*P_0_* < 0.05), but with no significant differences between groups P and H. During the overall experiment, the ADG and G/F values of piglets fed probiotics or antibiotics were higher than those of the negative control group N (*P_0_* < 0.05), and the piglets of groups P and H had a better growth performance than those of the other 3 groups. However, there was no significant difference in ADFI between the 5 groups during phases I, II and overall period. Furthermore, regression analysis indicated that the ADG and G/F have a remarkable linear relation with the dose of probiotics (*P_L_* < 0.05).

**Table 2:** Average daily gain (ADG), average daily feed intake (ADFI) and feed efficiency (G/F) of weaned piglets* fed with antibiotic growth promoters or *E. faecalis*.

**Item**	**N**	**P**	**L**	**M**	**H**	**SEM**	*P* value		
							***P_0_***	***P_L_***	***P_Q_***
Phase I (d 1to14)									
ADG, g	259^b^	282^a^	278^a^	277^a^	290^a^	5.03	0.013	0.045	0.444
ADFI, g	411	405	399	406	407	4.73	0.542	0.985	0.493
G/F, g/kg	631^b^	695^a^	696^a^	681^a^	712^a^	9.88	0.001	0.008	0.188
Phase II (d 15 to 28)									
ADG, g	364^d^	418^a^	379^cd^	392^bc^	406^ab^	5.10	<0.001	0.003	0.261
ADFI, g	695	721	709	719	716	17.21	0.814	0.581	0.565
G/F, g/kg	524^c^	579^a^	536^bc^	546^abc^	568^ab^	11.44	0.031	0.021	0.748
Overall (d 1 to 28)									
ADG, g	312^c^	350^a^	329^b^	335^b^	348^a^	3.26	<0.001	0.009	0.322
ADFI, g	553	563	555	563	562	8.28	0.829	0.619	0.742
G/F, g/kg	564^c^	621^a^	593^b^	595^b^	619^a^	6.79	<0.001	<0.001	0.134

*20 pens of piglets (18 piglets per pen) with pen as the experimental unit, and each mean based on 4 replicates.

N (negative control, basal diet); P (positive control, diet supplemented with neomycin); L, M, H (diets supplemented with probiotics 0.5×10^9^, 1.0×10^9^ and 2.5×10^9^ CFU/kg feed, respectively); SEM (standard error of the mean); *P_0_* (Statistical differences); *P_L_*, *P_Q_* (linear, and quadratic effect of 4 different doses of probiotics, respectively).

^a, b, c, d^ Mean values in the same row with different superscripts differ significantly (*P_0_* < 0.05).

Dietary treatments had no significant effect on diarrhea during phase I (*P_0_* > 0.05, Table 3), However, the diarrhea incidence reduced with the dose of probiotics increased (*P_L_* < 0.05). During phase II and the overall experiment, diarrhea incidence was reduced in piglets supplemented with antibiotics or probiotics compared to the negative control (*P_0_* < 0.05). Groups H and P had a lower diarrhea index than the other three groups, but there was no significant difference between groups H and P.

**Table 3:** Diarrhea incidence* of weaned piglets fed with neomycin or *E. faecalis*.

**Item**	**N**	**P**	**L**	**M**	**H**	**SEM**	*P* value		
							***P_0_***	***P_L_***	***P_Q_***
Phase I (d 1to14),%	4.2	2.9	3.8	3.3	2.3	0.45	0.075	0.010	0.860
Phase II (d 15 to 28),%	9.0^a^	4.2^b^	5.1^b^	4.0^b^	3.8^b^	0.41	<0.001	<0.001	<0.001
Overall (d 1 to 28),%	6.6^a^	3.6b^c^	4.4^b^	3.7^bc^	3.0^c^	0.35	<0.001	<0.001	0.001

N (negative control, basal diet); P (positive control, diet supplemented with neomycin); L, M, H (diets supplemented with probiotics 0.5×10^9^, 1.0×10^9^ and 2.5×10^9^ CFU/kg feed, respectively); SEM (standard error of the mean); *P_0_* (Statistical differences); *P_L_*, *P_Q_* (linear, and quadratic effect of 4 different doses of probiotics, respectively).

* Diarrhea incidence (%) = the total number of diarrheal piglets over the period divided by the number of piglets and days in the period multiplied by 100.

^a, b, c^ Mean values in the same row with different superscripts differ significantly (*P_0_* < 0.05).

### Blood Parameters

The contents of leukocytes, erythrocytes, and hemoglobin as well as the lymphocyte (%) were not affected by diet types (*P_0_* > 0.05; Table 4). However, the four groups (P, L, M, and H) were numerically lower than group N in leukocyte concentration. Furthermore, the mean corpuscular hemoglobin concentration was higher in group N than in groups P and H (*P_0_* < 0.05. The average platelet of the probiotics groups increased along with the dose of probiotics (*P_L_* < 0.05), and the platelet content was higher in group H than group N (*P_0_* < 0.05).

**Table 4:** Blood parameters of weaned piglets* fed diets with neomycin or *E. faecalis*.

**Item**	**N**	**P**	**L**	**M**	**H**	**SEM**	*P* value		
							***P_0_***	***P_L_***	***P_Q_***
Leukocytes, 10^3^/mm^3^	37.2	27.2	29.6	28.0	28.1	1.58	0.252	0.170	0.176
Erythrocytes, 10^6^/mm^3^	6.26	6.75	6.49	6.15	6.65	0.11	0.348	0.311	0.548
Hemoglobin g/dl	10.5	10.8	10.3	10.6	10.4	0.12	0.810	0.922	0.747
MCHC	288^a^	270^bc^	277^ab^	278^ab^	265^c^	2.31	0.004	0.002	0.658
Platelet, 10^3^/mm^3^	564^b^	688^ab^	516^b^	649^b^	849^a^	32.1	0.006	<0.001	0.364
Lymphocyte, %	57.3	61.5	65.2	60.7	55.9	2.10	0.672	0.534	0.409

* Blood samples were taken from 5 weaned piglets per treatment.

N (negative control, basal diet); P (positive control, diet supplemented with neomycin); L, M, H (diets supplemented with probiotics 0.5×10^9^, 1.0×10^9^ and 2.5×10^9^ CFU/kg feed, respectively); MCHC (mean corpuscular hemoglobin concentration); SEM (standard error of the mean); *P_0_* (Statistical differences); *P_L_*, *P_Q_* (linear, and quadratic effect of 4 different doses of probiotics, respectively).

^a, b, c^ Mean values in the same row with different superscripts differ significantly (*P_0_* < 0.05).

### Bacterial Community Based on PCR-DGGE

**Figure 1:**
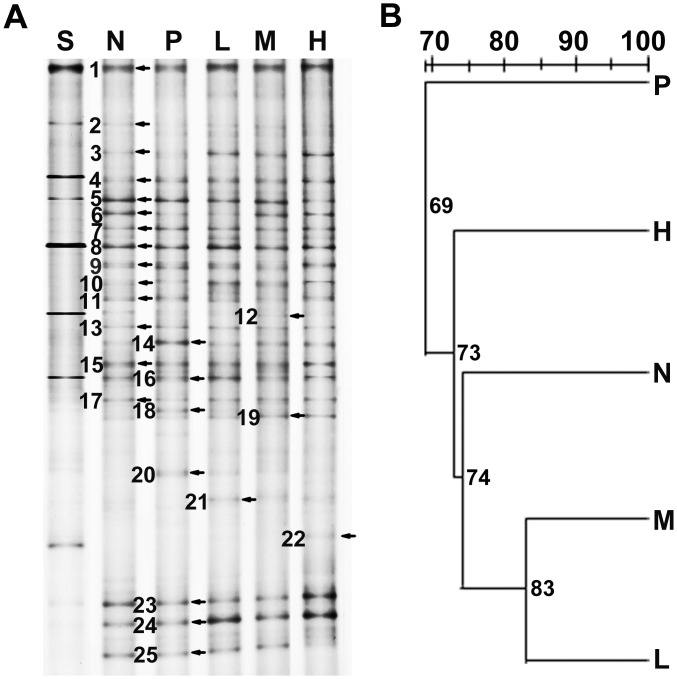
Bacterial community of weaned piglets fed with neomycin or *E. faecalis*. (A) DGGE profiles of the V6~V8 regions of the 16S rDNA gene fragments from the samples. The denaturant gradient range is from 42% to 58%. The major difference bands are numbered. Lane S (Standard ladder, which are PCR products generated from different bacterial 16S rDNA genes with primers 968F-GC and 1401R); N (negative control, basal diet); P (positive control, diet supplemented with neomycin); L, M, H (diets supplemented with probiotics 0.5×10^9^, 1.0×10^9^ and 2.5×10^9^ CFU/kg feed, respectively); (B) UPGMA cluster analysis of Dice similarity indices from DGGE profiles.

The data of the bacterial communities revealed a high degree of variation between the samples (Fig. 1). There were some changes in the relative intensity of dominant bands (Fig. 1A) and an 83% similarity in the bacterial community structures of groups L and M (Fig. 1B). As shown in Table 5, in terms of the species richness (as estimated by the number of major bands present in DGGE profiles), groups L and M were numerically higher than groups N, P and H. As for the Shannon index H′ for the bacterial community, group M (2.98) had a higher bacterial diversity than the others, and group N had the lowest value (2.81). For the Simpson index (1/D), the results of bacterial diversity were similar to those of H′. The results showed that the medium dose of probiotics (M) was more effective than the low and high doses of probiotics (L and H) in improving fecal bacterial diversity of piglets.

**Table 5:** Bacterial diversity index calculated from the DGGE banding patterns (Fig. 1A).

**Index**	**N**	**P**	**L**	**M**	**H**
Species richness (S)	23	23	25	27	23
Shannon index (H′)	2.81	2.86	2.84	2.98	2.82
Evenness (J)	0.90	0.91	0.88	0.91	0.90
Simpson index (1/D)*	13.61	15.07	12.97	15.91	13.15

N (negative control, basal diet); P (positive control, diet supplemented with neomycin); L, M, H (diets supplemented with probiotics 0.5×10^9^, 1.0×10^9^ and 2.5×10^9^ CFU/kg feed, respectively);

*1/D, the reciprocal of Simpson diversity index.

A total number of 25 dominant bands in the DGGE profiles (marked with numbers in Fig. 1A) were sequenced, and the relative identification is reported in Table 6. The sequence similarity of all bands was ≥ 96% as compared with those available in GenBank database. *Clostridium* sp., *Robinsoniella* sp., *Ruminococcus* sp., *Eubacterium* sp., *Faecalibacterium* sp., *Lactobacillus* sp. and some uncultured bacteria were found in the samples. The major difference bands shown in Fig. 1A are bands 3, 6, 14, 18, 20 and 21. In lane P (the antibiotics group), the relative intensities of bands 3 (*Eubacterium eligens*), and 6 (uncultured bacterium) decreased, and band 21 (*F. prausnitzii*) disappeared, but those of bands 14 (uncultured bacterium), 18 (*Ruminococcus obeum*), and 20 (uncultured *Lachnospiraceae* bacterium) increased. In probiotics groups, a new band related to *F. prausnitzii* was found as compared to the control groups.

**Table 6:** Identification of band fragments in DGGE gels (Fig. 1A).

Band no. *	**Closest relative and NCBI accession number**	**Identity^◆^**
1	Uncultured bacterium (JQ187036.1)	99%
2	*Clostridium mayombei* DSM 6539T (FR733682.1)	100%
3	*Eubacterium eligens* ATCC 27750 (CP001104.1)	97%
4	Uncultured bacterium (NR_025207.1)	97%
5	*Robinsoniella peoriensis* (AF445283.2)	96%
6	Uncultured bacterium (HQ716245.1)	100%
7	*Ruminococcus* sp. (FJ611794.2)	100%
8	*Clostridium irregulare* (JX898025.1)	97%
9	Uncultured bacterium (JQ820130.1)	100%
10	Clostridiaceae bacterium (EU728782.1)	98%
11	*Eubacterium coprostanoligenes* (HM037995.1)	99%
12	Uncultured bacterium (GQ137884.1)	99%
13	Swine manure bacterium (AY167964.1)	99%
14	Uncultured bacterium (FP077070.1)	100%
15	Uncultured bacterium (FP078153.1)	99%
16	Uncultured *Firmicutes* bacterium (JN568109.1)	100%
17	*Lachnospiraceae* bacterium (EU728751.1)	99%
18	*Ruminococcus obeum* (AY169411.1)	100%
19	Uncultured bacterium (EU472437.1)	97%
20	Uncultured *Lachnospiraceae* bacterium (JX230492.1)	100%
21	*Faecalibacterium prausnitzii* (AY169430.1)	99%
22	Uncultured bacterium (HF952852.1)	99%
23	*Lactobacillus reuteri* (CP006603.1)	99%
24	*Lactobacillus kitasatonis* DSM 16761T (FR683090.1)	98%
25	*Lactobacillus johnsonii* NCC 533 (NR_075064.1)	96%

* Bands are numbered according to Fig. 1A.

^◆^Identity represents the sequence identity (%) compared with that in the GenBank database.

### *Lactobacillus* Copy Numbers and Community

The quantification of gene targets encoding 16S rDNA gene sequences from the fecal samples revealed that the relative number of *Lactobacillus* increased with the dose of probiotics(*P_L_* < 0.05, Table 7), and groups H and P had a significantly higher relative number of *Lactobacillus* than the negative control N (*P_0_* < 0.05, Table 7). However, no significant difference was found in the number of total bacteria (*P_0_* > 0.05, Table 7).

**Table 7:** qPCR analysis of the copy numbers total bacteria and relative number of *Lactobacillus* *.

**Item**	**N**	**P**	**L**	**M**	**H**	**SEM**	*P* value		
							***P_0_***	***P_L_***	***P_Q_***
Total bacteria (log^10^ copies/g)	10.58	10.31	10.30	10.34	10.36	0.05	0.313	0.366	0.122
*Lactobacillus*, %	15.62^c^	25.25^ab^	15.10^c^	19.26^bc^	26.75^a^	1.29	0.002	<0.001	0.531

* Fecal samples were taken from 5 weaned piglets per treatment.

N (negative control, basal diet); P (positive control, diet supplemented with neomycin); L, M, H (diets supplemented with probiotics 0.5×10^9^, 1.0×10^9^ and 2.5×10^9^ CFU/kg feed, respectively); SEM (standard error of the mean); *P_0_* (Statistical differences); *P_L_*, *P_Q_* (linear, and quadratic effect of 4 different doses of probiotics, respectively).

^a, b, c^ Mean values in the same row with different superscripts differ significantly (*P_0_* < 0.05).

**Figure 2:**
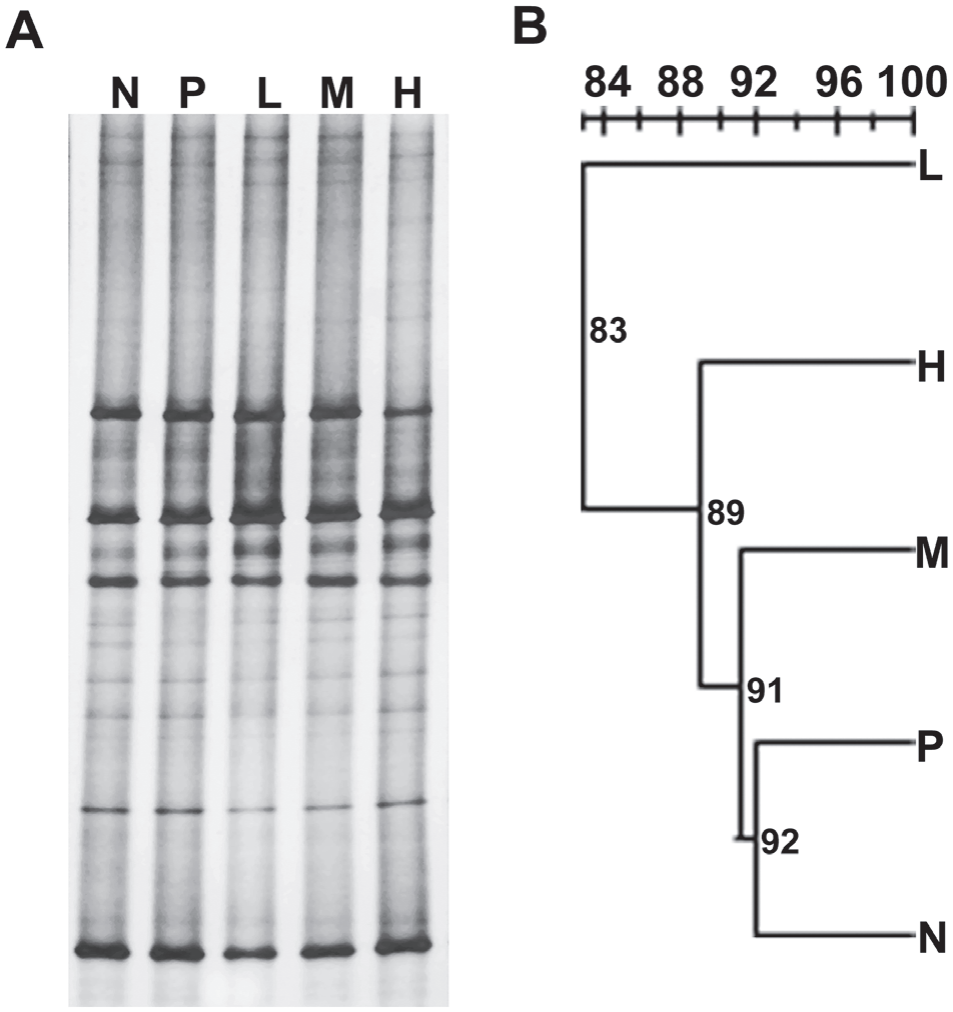
*Lactobacillus* community of weaned piglets fed with neomycin or *E. faecalis*. (A) DGGE profiles of V3 region of the 16S rDNA gene fragments with the primes Lac1 and Lac2-GC. The denaturant gradient range is from 41% to 60%. Lanes N (negative control, basal diet); P (positive control, diet supplemented with neomycin); L, M, H (diets supplemented with probiotics 0.5×10^9^, 1.0×10^9^ and 2.5×10^9^ CFU/kg feed, respectively); (B) UPGMA cluster analysis of Dice similarity indices from DGGE profiles.

No significant difference was shown in *Lactobacillus* composition and diversity by PCR-DGGE (Fig. 2, Table 8). However, the Shannon index (H′) and Simpson index (1/D) of groups L, M and H showed a slightly higher *Lactobacillus* diversity than groups N and P. As shown in Fig. 2A, the relative intensity of the dominating bands and the total number of bands in each lane were similar in all 5 groups, with fewer bands in lane P (11 bands) and more bands in lane H (15 bands). The UPGMA cluster analysis showed that groups P and N had a 92% similarity in the *Lactobacillus* community structure (Fig. 2B).

**Table 8:** *Lactobacillus* diversity index calculated from the DGGE banding patterns (Fig. 2A).

**Index**	**N**	**P**	**L**	**M**	**H**
Species richness (S)	13	11	12	13	15
Shannon index (H′)	2.03	2.00	2.09	2.08	2.15
Evenness (J)	0.79	0.84	0.84	0.81	0.80
Simpson index (1/D)*	6.06	6.20	6.77	6.50	6.71

N (negative control, basal diet); P (positive control, diet supplemented with neomycin); L, M, H (diets supplemented with probiotics 0.5×10^9^, 1.0×10^9^ and 2.5×10^9^ CFU/kg feed, respectively);

*1/D, the reciprocal of Simpson diversity index.

## Discussion

Lactic acid bacteria (LAB) are found as commensals in the animal gastrointestinal tract and have a long history of safety and demonstrable beneficial properties. However, the effect of the LAB *E. faecalis* on the diversity of total bacteria and *Lactobacillus* is not well understood. We suppose that *E. faecalis* LAB31 benefits the balance of intestinal communities by stimulating the growth of beneficial microbes. Once the intestinal health is improved, the diarrhea incidence will be reduced, thus improving the growth performance of the host. We combined DGGE and qPCR to study the fecal total bacteria and *Lactobacillus* spp. of piglets. DGGE has been used successfully to study the taxonomy of the bacterial community in pigs [28], [32], while qPCR was used to investigate the precise and profound changes in the abundance of *Lactobacillus* group.

Weaning is a stressful period in the life of piglets, which is associated with changes in diet, gut environment and morphology [33], and thus may result in a low growth rate, diarrhea, imbalanced intestinal microbiota and impaired health. These problems can be reduced and the performance of the host can be improved by supplementing the diet with probiotics. In this study, diets supplemented with *E. faecalis* LAB31 improved the growth performance of piglets, and the high dose (H) was better than the medium (M), and low (L) doses of probiotics. Mallo et al. reported that supplementation of a probiotic *E. faecium* at a dose of 10^9^ CFU/kg to the diet of pigs improved their growth and feed conversion [13]. Similar results were reported for the application of *E. faecium* probiotics to pigs by Shidara et al. [12] and Marcin et al. [34].

As we know, diarrhea has a significant impact on gut health and growth performance of piglets. The statistics indicated that the piglets supplemented with neomycin or probiotics reduced the diarrhea incidence, suggesting that *E. faecalis* LAB31 could effectively prevent diarrhea in weaned piglets. Several previous studies reported that supplementation with *E. faecium* reduced the number of piglets suffering from diarrhea [35], [36]. Another study even demonstrated that the complex *Lactobacillus* preparation derived from the digestive tract of a healthy weaning pig can prevent the diarrhea challenged by pathogenic *E. coli* and increase the post-weaning performance of pigs [37]. Assuming that *E. faecalis* LAB31 might affect the intestinal microbiota of piglets associated with diarrhea, the fresh fecal samples of piglets were collected for further microbial analyses.

The species richness of the samples is usually represented by the number of identifiable DGGE bands. In this study, the number of bands and bacterial diversity increased numerically in probiotic group M. Taras et al. demonstrated that the bacterial communities of probiotic-treated pigs could be modified by application of *E. faecium* based on DGGE analysis [38]. Furthermore, DGGE demonstrated that piglets supplemented with *Lactobacillus plantarum* significantly increased the Simpson’s index in gastrointestinal tract microbial communities [39]. A higher bacterial diversity could represent a benefit for the weaned animals because of the possible link between the diversity of ecosystems and their ability to respond to perturbations [40], indicating that bacterial diversity can aid host health and limit bacterial pathogen colonization [41].

Most species identified from the DGGE profiles are uncultured bacteria, and belong to *Firmicutes*. Many other studies have found that *Firmicutes* such as the *Clostridium leptum* subgroup constitute a large portion of the fecal community of pigs [42], [43]. The 16S rDNA gene sequences of some major differential bands were related to *Eubacterium eligens*, *Faecalibacterium prausnitzii* and *Ruminococcus obeum*. Finished genome sequences were generated from *E. rectale* and *E. eligens*, belonging to Clostridium Cluster XIVa, one of the most common gut *Firmicute* clades, which includes many species producing butyrate from either acetate or lactate [44], [45]. *Ruminococcus* species were found to be present as active bacteria and could be important members of the bacterial community in the pig gastrointestinal tract [46], [47]. Interestingly, a band related to *F. prausnitzii*, which is one of the most abundant commensal bacteria in the human intestinal microbiota of healthy adults, was detected in the probiotics groups [48]. Also, *F. prausnitzii* is widely distributed in the hindgut of pigs and other mammals [49]. More importantly, some studies suggest that *F. prausnitzii* is an anti-inflammatory bacterium with therapeutic potential for mice and humans with inflammatory bowel disease [50], [51], [52]. However, further study is needed to determine the relation between *F. prausnitzii*-like bacteria and intestinal health of pigs.

The most diverse genus of LAB is Lactobacilli, which are commonly found in humans and animal gastrointestinal tract, and play an important role in intestinal health [53]. Moreover, lactobacilli are regarded as the dominant LAB and assumed to play a major role in the pig intestine, while bifidobacteria are present in lower amounts and to a lesser extent [54], [55]. In this study, DGGE analysis showed that some dominant bands were related to *Lactobacillus* spp., such as *L. reuteri*, *L. kitasatonis*, and *L. johnsonii*. This result is similar to some previous studies which have found some species of *Lactobacillus* in the intestinal samples of piglets, such as *L. sobrius* and *L. delbrueckii* in small intestine and *L. sobrius* in colon [39], as well as *L. johnsonii* and *L. reuteri* in the hindgut [56].

*Lactobacillus* species are regarded as beneficial bacteria in gastrointestinal tract, and are important for a well-balanced intestinal microbiota because of their health-promoting effects such as prevention of diarrhea and intestinal infections. Therefore, *Lactobacillus* spp. was selected for qPCR investigation, and the results showed that piglets supplemented with neomycin or probiotics significantly increased the relative number of *Lactobacillus*. However, the total bacteria were not significantly affected by the application of neomycin or probiotics. These results indicated that the growth of *Lactobacillus* spp., compared to the other bacteria, was promoted by supplementation of neomycin or probiotics. It is known that neomycin is an aminoglycoside antibiotic, which can inhibit a wide range of gram-negative bacteria and some gram-positive bacteria, such as *Enterobacter* spp., *Escherichia* spp., and *Proteus* spp. However, most of the lactobacilli were resistant to aminoglycosides [57]. Furthermore, the relative number of *Lactobacillus* increased with the dose of probiotics. The bacterial inhibition test in vitro showed that *E. faecalis* LAB31 possessed activity against gram-negative bacteria, such as *E. coli* (O139, C83905, C83524, C83529) and *Salmonella typhimurium* O4 Hi [58]. Considering the increase of the relative number of *Lactobacillus* from the inhibition of some bacteria, *E. faecalis* LAB31 may have the similar function in vivo. However, this hypothesis remains to be elucidated in the future. Some studies also found that supplementation of *E. faecium* and *E. faecalis* increased fecal *Lactobacillus* concentration in pigs [59], and in broiler chicken [60], but decreased other bacteria such as *Streptococcus* spp., *Clostridia* and coliforms in humans [61]. Additionally, the high copy number percentage of lactobacilli can be ascribed to the limitations of primers and qPCR.

In order to obtain the composition and diversity of *Lactobacillus*, a number of 340-bp fragments of the V3 region of the 16S rDNA gene were amplified by PCR with primers Lac1 and Lac2GC. This sequence represented most of the *Lactobacillus* species to be differentiated, and could be used in large-scale *Lactobacillus* community investigations of the intestinal tract of humans and other animals [29]. A previous study demonstrated that the composition of colonic *Lactobacillus* was altered in piglets by oral administration of *Lactobacillus sobrius* S1 based on DGGE analysis [56]. However, diets supplemented with *E. faecalis* LAB31 had no significant effects on the *Lactobacillus* composition of piglets. It can be concluded that there was a stable proportion between dominant *Lactobacillus* species and total *Lactobacillus* spp. in feces. The results of qPCR and *Lactobacillus*-specific PCR-DGGE indicated that the relative numbers of *Lactobacillus* were increased, while the *Lactobacillus* composition was not affected by supplementation of neomycin or *E. faecalis* LAB31.

The effect of probiotics on the immune system has attracted the attention of researchers only in recent years. In this study, no significant differences were detected in leukocytes, erythrocytes, hemoglobin, and lymphocyte (%). Some studies reported that pigs supplemented with *E. faecium* or other probiotics showed no obvious immune-stimulatory effect [62], or effects on blood parameters of leukocytes, erythrocytes and lymphocyte [63]. However, the addition of *E. faecalis* LAB31 significantly increased the platelet count, and reduced the MCHC compared to the control N. These results remain to be elucidated because few references are available to support them. Furthermore, several studies indicate that the population of circulating and intraepithelial T cells [64], and IgA [65] are affected by the application of LAB in piglets. Perhaps, further research is required to evaluate some other parameters associated with the immune system such as IgG, IgA and IgM.

In conclusion, the *E. faecalis* LAB31 effectively improved the growth performance and reduced the incidence of diarrhea in weaned piglets by altering the bacterial fecal community and increasing the relative number of *Lactobacillus* in the feces of weaned piglets. The results suggest that *E. faecalis* LAB31 can serve as an alternative to antibiotics in diets for weaned piglets.

## Supporting Information

S1 Table Ingredient and chemical composition of basal diet. (DOC)
