# Supplementary material for: Dietary Enterococcus faecalis LAB31 Improves Growth Performance, Reduces Diarrhea, and Increases Fecal Lactobacillus Number of Weaned Piglets
Source: PLoS One. 2015 Jan 24;10(1):e0116635. doi: 10.1371/journal.pone.0116635 (PMC4305361; doi:10.1371/journal.pone.0116635)
Supplement: S1 Table — (DOC) [file pone.0116635.s001.doc]

**Table S1.** Ingredient and chemical composition of basal diet.

| Item | Phase I (d 1to14) | Phase II (d 15 to 28) |
| --- | --- | --- |
| Ingredient,% |  |  |
| Corn, yellow | 47.59 | 60.13 |
| Soybean meal, 43% CP | 10.00 | 16.00 |
| Spray dried plasma protein | 3.50 |  |
| Fish meal | 5.00 | 5.00 |
| Dried whey | 26.50 | 14.00 |
| Dicalcium phosphate | 1.05 | 1.40 |
| Limestone | 0.15 | 0.15 |
| L-Lysine-HCL (98%) | 0.39 | 0.43 |
| DL-Methionine (99%) | 0.17 | 0.14 |
| Sugar | 3.00 |  |
| Salt | 0.20 | 0.30 |
| [Corn](app:ds:corn) [starch](app:ds:flour) | 1.40 | 1.40 |
| Vitamin/mineral premix^*^ | 1.05 | 1.05 |
| Chemical composition |  |  |
| ME, kcal/kg） | 3645 | 3458 |
| CP (%) | 20.5 | 19.0 |
| Lys (%) | 1.60 | 1.48 |
| Met (%) | 0.91 | 0.80 |
| Ca (%) | 0.69 | 0.59 |
| P (%) | 0.50 | 0.45 |

Dietary treatments: N (negative control, basal diet without antibiotics or probiotics); P (positive control, diet supplemented with 100 mg/kg of neomycin sulfate); L, M, H (diets supplemented with probiotics 0.5×10^9^, 1.0×10^9^ and 2.5×10^9^ CFU/kg feed, respectively); Antibiotic and probiotic products were added to the diets at the expense of corn starch.

^*^Provided the following per kg of diet: 12,800 IU of vitamin A, 4,000 IU of vitamin D_3_, 80 mg of vitamin E, 4 mg of vitamin B_1_, 10 mg of vitamin B_2_, 6 mg of vitamin B_6_, 46 μg of vitamin B_12_, 4 mg of vitamin K_3_, 20 mg of pantothenic acid, 40 mg of nicotinic acid, 0.36 mg of biotin, 2 mg of folic acid, 500 mg of choline chloride; 80 mg of Mn, 200 mg of Fe, 40 mg of Cu, 120 mg of Zn, 0.4 mg of I, 0.25 mg Co, and 0.4 mg of Se.
